# “Exposing force”: the COVID-19 pandemic and women's sport in Ireland during 2020–2021

**DOI:** 10.3389/fspor.2023.1196184

**Published:** 2023-07-20

**Authors:** Seán Crosson, Maedhbh Ní Chumhaill

**Affiliations:** ^1^ Huston School of Film & Digital Media, University of Galway, Galway, Ireland; ^2^ Independent Researcher, Galway, Ireland

**Keywords:** COVID-19, pandemic, Ireland, women’s sport, gaelic games, sport media, sport funding

## Abstract

**Introduction:**

An analysis of how the pandemic served to highlight neglected weaknesses and inequalities with regard to the structures and supports available to facilitate women's sport in Ireland.

**Methods:**

A survey conducted in the summer of 2021 with 194 female athletes across the island of Ireland. These athletes were engaged with the sports of camogie, Ladies Gaelic football, hockey, and rugby, and each responded to a 28-question survey.

**Results:**

Our findings indicate that the experiences of female athletes during the COVID-19 pandemic raise serious questions regarding equality in sport across gender lines. Concerns expressed by the surveyed athletes, especially in relation to access to facilities, inadequate sponsorship, and funding reveal salient aspects of the experiences of Irish female athletes during the pandemic, and its role as an “exposing force” of inequalities within Irish sport.

**Conclusion:**

The COVID-19 pandemic in Ireland has been a challenging and revealing period with regard to sport in Ireland, acting as an “exposing force” of the existing inequitable structures for both the support and coverage of women's sport. Such challenging periods can also offer opportunities to learn, progress and improve and already in the past year there is evidence of positive developments for women's sport in Ireland, developments that we contend were expedited due to the impact of the COVID-19 pandemic in Ireland.

## 1. Introduction

The COVID-19 pandemic revealed structural and ongoing issues with regard to the manner in which women's sports are facilitated and promoted internationally, including in Ireland. *Irish Times* journalist Una Mullally (1) argues that the pandemic acted as an “exposing force” that highlighted the inadequacies of health and other organisational infrastructures, services and supports in an Irish society weakened by years of neoliberal orthodoxy. It exposed shortcomings dating back decades, but that had become particularly exacerbated following the 2008 global financial crisis and the austerity measures that ensued. Here we borrow the term “exposing force” to trace how the pandemic served to highlight neglected weaknesses and inequalities with regard to the structures and supports available to facilitate women's sport in Ireland compared to men's equivalent sports. Our findings are primarily informed by a survey conducted in June and July of 2021 with 194 female athletes across the island of Ireland, but they are in line with broader findings made internationally regarding the unequal treatment of female athletes during the COVID-19 pandemic [including (2–6)]. The athletes surveyed were principally engaged with the indigenous sports of camogie and Ladies Gaelic football, popular team-sports played by women and girls in Ireland (though a small cohort were involved with hockey and rugby), and each responded to a 28-question survey. Respondents were asked about their experiences of playing sport during the pandemic, and their perception of the position of women's sport in Ireland, both prior to the pandemic and at the time the survey was undertaken.

As in other societies across the world, women's engagement with sport was slow to develop and often discouraged for much of the 19th and early 20th century in Ireland. At the turn of the 20th century the “hobby sports” for women were allowed when their other expected and highly gendered commitments in society were met. In time, women's sport moved from a harmless novelty, where sporting endeavors were belittled or diminished, to an activity that was sometimes harshly criticized (7). It would be in the latter half of the 20th century and into the 21st before women's sport began to receive appropriate facilitation and recognition, though our findings indicate ongoing challenges and inequalities that remain to be addressed.

## 2. Methods

In the summer of 2021, 194 athletes across the island of Ireland responded to a 28-question online survey. The survey focused primarily on athletes who played camogie and/or Ladies Gaelic football (which 193 of the respondents were engaged with), though a small number that also played rugby and/or hockey also responded. The survey was posted into team group chats, primarily circulated through WhatsApp, and relevant ethical protocols were followed, including a requirement that all respondents were over 18, were fully informed of the purpose of the survey, gave their consent to participate, and that no personal identifying information was requested or recorded as part of this process. While individuals engaged with both hockey and rugby were among respondents, the principal focus was on players of the amateur Gaelic games of camogie and Ladies Gaelic Football as popular indigenous team sports engaged in by women and girls in Ireland. Ladies Gaelic Football and camogie are administered respectively by the Ladies Gaelic Football Association (LGFA) and the Camogie Association (CA), distinct and independent associations from the larger Gaelic Athletic Association (GAA) that administers Gaelic games for men and boys in Ireland. However, both associations are dependent on the GAA for access to playing facilities while individual Gaelic games clubs and county boards (the elite level of the sport) usually have both male and female teams, though each are administered by separate associations. These distinctions are important to note at the outset as they were contributory factors in the experiences of female athletes detailed below.

The survey was live from 29 June 2021 until 22 July 2021. During this time athletes from 27 counties and all provinces in Ireland shared their perspectives anonymously (safeguards were put in place to ensure the anonymity of all respondents^1^). Other sports that respondents to the survey played included handball, basketball, equestrianism, gymnastics, soccer, tennis, badminton, surfing, swimming, show jumping, kayaking, athletics, golf and Muay Thai.

In May of 2021 the survey was piloted with a small test group consisting of players, sport administrators and coaches based in counties Roscommon, Clare, Dublin and Tipperary. Their feedback allowed for further analysis and discussion of the design and structure of the research tool to ensure that it was effective, clear, and fit for purpose. This ensured the validity, integrity, and overall effectiveness of the survey approach. The survey was designed to gather information on the impact of the pandemic, the gender dynamics in clubs, and players' experiences of funding, media coverage, recognition within their sport, and overall support.

The survey was divided into four different sections and on average took 6 min 53 s to complete. Hosted on Microsoft forms, the survey questions included open-ended, sliding scale, multiple choice, and binary yes/no questions. After an initial introduction (Section 1), Section 2 featured questions asking which sport(s) athletes played, where they played, and whether they would be considered an elite athlete or not. Respondents were asked how they were affected by the pandemic and were given a series of options to select. In Section 3, athletes responded to a series of statements about different aspects of women's sport in Ireland “currently” (June–July 2021) and “prior” to the pandemic. Athletes were asked about the barriers in returning to play; if the clubs they played for had male and female teams; and (if this was the case) a series of questions were asked about the treatment they receive in their club(s), in comparison to their male counterparts. The second last question in the survey (Q. 26) asked: “As we emerge from the pandemic do you believe women's sport (its progression and support) will be in a better position” and respondents were encouraged to elaborate on their response to this question (our discussion below is largely informed by these responses). The final section (Section 4) thanked respondents for their participation and encouraged them to share the survey with other female athletes.

## 3. Results and discussion

**Figure 1:**
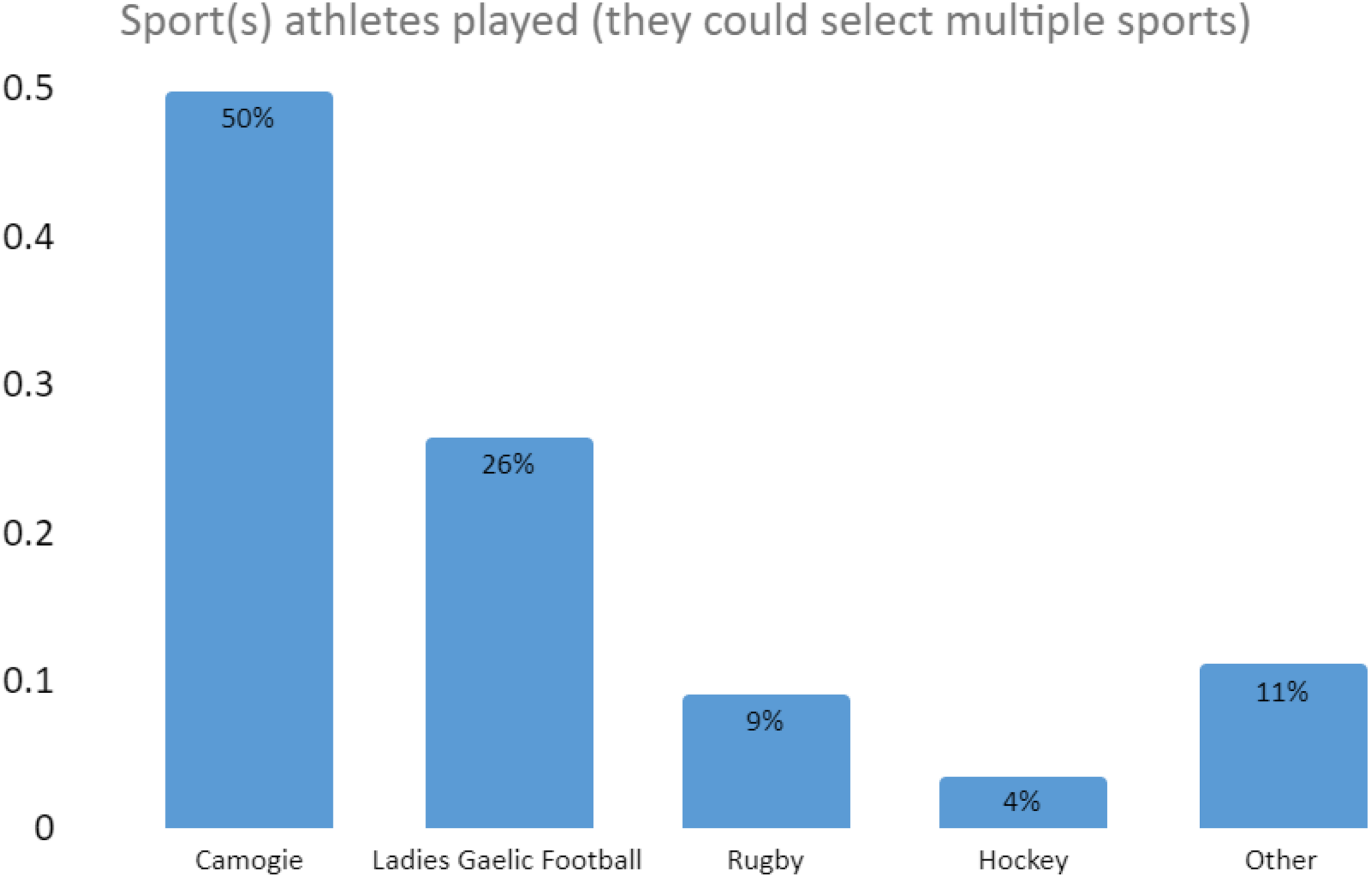
Sports engaged with by respondents (multiple sports could be chosen).

Figure 1 below indicates the range of sports played by respondents to the survey. The majority of our respondents were engaged with Gaelic Games as this was the principal focus.

**Figure 2:**
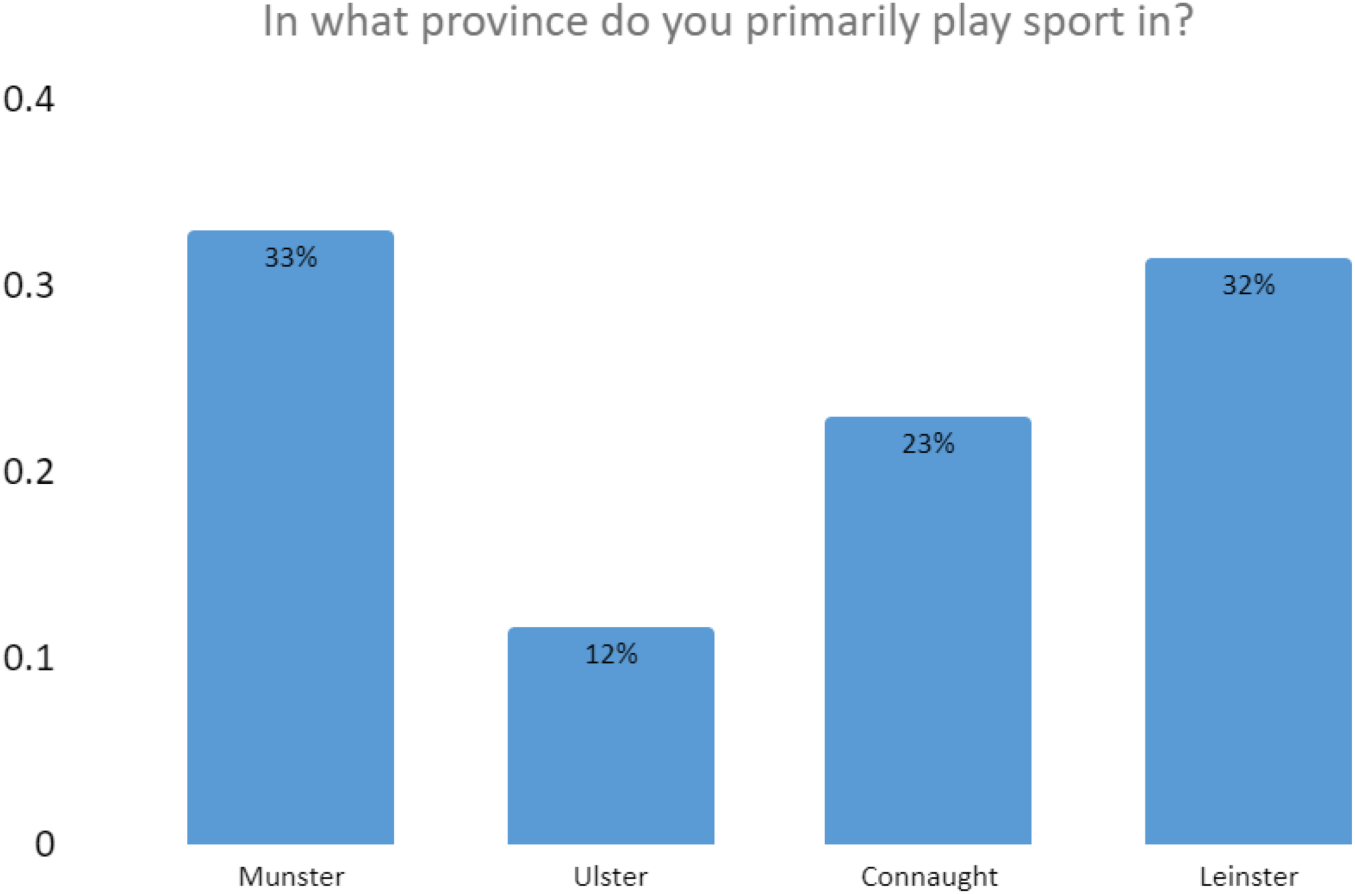
Geographical spread of respondents.

28% of our respondents were engaged with elite level sport in Ireland. Elite athletes are players that play at inter-county level for Gaelic games or are on the senior teams for hockey or rugby, with the majority of our respondents (72%) engaged with sport at a lower level, usually through a local club. Figure 2 indicates the geographical spread of respondents.

Table 1 indicates the responses to our question enquiring how athletes were “impacted by the pandemic”, with most respondents indicating a negative impact on their participation, performance or overall physical or mental wellbeing.

**Table 1:** How were respondents impacted by the pandemic.

Impact of the pandemic	Response %^a^
Change in fitness/skill level	21%
Cancelled matches/tournaments/competitions	23%
Player number restrictions at training	12%
Player contact restrictions at training	14%
Virtual training	14%
Impact on mental health	15%
Other	1%

^a^ Please note athletes could select multiple responses.

In question 6 in Section 2, respondents could provide further relevant detail regarding their experiences during the pandemic. Respondents noted an impact on their motivation, teammates quitting, restrictions in travel to training, injury due to a lack of training over a prolonged period, an increase in anxiety levels, and missing the social aspect of seeing teammates and friends.

**Figure 3:**
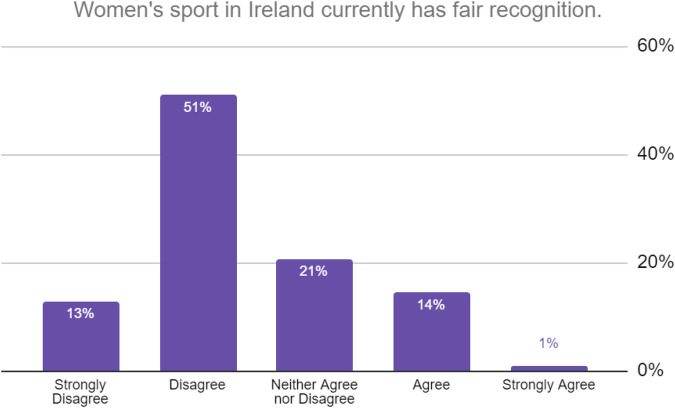
Responses to question “to what extent do you agree or disagree with the following statement.”.

64% of respondents did not believe that women's sport in Ireland had fair recognition during the pandemic (See Figure 3). Similar concerns were evident from respondents with regard to sponsorship or funding available to women's sport in Ireland during the pandemic with 75% disagreeing (or strongly disagreeing) with the statement that “women's sport in Ireland has sufficient sponsorship/funding”. Respondents were also asked their opinion with regard to media coverage during the pandemic, and again over 75% disagreed or strongly disagreed with the statement that “women's sport in Ireland had adequate media coverage.” However over 50% of respondents (despite the concerns raised in responses to earlier questions) agreed or strongly agreed that women's sport “is currently in a positive position” and 88% were happy with the return to play process after the pandemic.

Of the 12% who indicated unhappiness with the return to play process, some players still had yet to return to play at all at the time of the survey (July 2021). This applied particularly to indoor sports such as handball and basketball, which were not the principal focus of this study. The main barriers to return to play indicated by respondents included access to facilities, lockdown restrictions and decrease in fitness/skills levels. Players noted that pandemic regulations were inconsistent and confusing. They were frustrated by poor communication regarding fixtures, guidelines for clubs and counties, and that clarity on these issues was provided too late. One player noted that it was harder for women's teams in her club to acquire a COVID-19 officer (8)^2^ to ensure that training could go ahead. Some players also expressed a lack of enjoyment at training as training sessions seemed rushed and there was a scarcity of training slots.

95% of respondents indicated that their club had both male and female teams. However, as noted above, while female teams may have represented the same club as their male counterparts, they were not affiliated to the Gaelic Athletic Association (GAA, whose facilities were been used)—the GAA hired out their facilities to the associations administering female sports (Ladies Gaelic Football Association and the Camogie Association), but these associations do not own their own facilities, and this had consequences for the prioritisation of access to facilities. Those who played in clubs with men's and women's teams were asked questions pertaining to the treatment of the women's teams in their club compared to the men's teams in their club and almost 60% of respondents did not view men's and women's teams as being treated fairly and equally. The majority of respondents expressed serious concerns regarding the inequitable and unfair treatment they felt women's teams received, whether in terms of access to pitches and other club facilities (e.g., gyms) (57%), overall support from the club (84%), and the prioritisation of men's teams (84%).

Overall, the responses provided to the survey reveal COVID-19 as an exposing force of inequalities with regard to the experience of male and female participants engaged with sport. Female athletes shared significant concerns regarding the unequal treatment of girls and women's sports during the pandemic and in the return to play process. Descriptive answers from the open-ended questions were analysed and distinct themes became apparent, in particular around access to facilities and resourcing, media coverage, funding, and the longer-term consequences of the pandemic, and the following sections provide a summary overview of these themes.

### 3.1. Access to facilities and resourcing

The disparity in treatment of players in comparison to their male counterparts in their clubs and on a national level was communicated throughout survey responses. A statement from one player serves as a crucial insight into the shared experience of many athletes that responded to the survey. While disagreeing with the question “Currently do you think women's sport in IRELAND is in a positive position?”, she elaborated with the response below

“Less funding, less recognition for effort, less facilities at club level and less financial means at club and county level, less people at matches, less general support, constant comparison to men's sport, failure to accept and appreciate female sport as valid in and of itself, less media coverage to an appalling degree and even when recently pointed out it continues to be a constant issue. One front or back cover every few weeks does not make up for 11.5 of 12 pages covering men's sport”—Camogie (non-elite) Clare

55% of respondents did not believe that women's teams were treated fairly and equally in their club, with unequal access to the same quality pitches a matter of considerable concern, as indicated in the comment below:

 “Significantly… men are given priority with regards [to] venue, match fixtures, facilities.”—Camogie (non-elite) Roscommon

Respondents indicated that priority in terms of pitch access, scheduling and access to club facilities such as gym and flood-lights was afforded to men and boy's teams. This affected the ability of female teams to train and impinged on their overall skill and fitness with 21% of respondents noting a change in skill and fitness level as a significant impact of the pandemic and their ability to return to play.

Significantly 84% of respondents answered “yes” when asked “have you experienced or seen men's sports teams given priority over women's teams?” Out of the 162 respondents that responded in this manner, 87 explicitly mentioned limitations on access to pitches and other club facilities such as the club gym:

“Younger boys age groups being given the main pitch for training over our older ladies team. Yes I think it has increased [since the pandemic] as there is way more demand for the pitches and they always are given first choice’—Camogie/Ladies Gaelic Football (non-elite) Clare

Respondents cited scheduling as an increased issue between men's and women's teams in their club, indicating that the norm was for men's teams to get first choice when it came to training slots and pitch use. According to some respondents, men's teams (in their clubs) were also afforded the advantage of more convenient playing times:

“Games/training moved due to changes in schedules to [the] men's team. Priority given to men's teams”—Camogie (elite) Clare

While players noted that they were given less access than their male counterparts to club facilities they also mentioned that in certain circumstances public health guidelines were used as an excuse to limit the access female players had to equipment and facilities. Some respondents noted that the same rules were not applied to men's teams in the same clubs. As noted already, a crucial contributing factor here is that neither the LGFA nor CA own the facilities concerned and must rely on the generosity of GAA clubs that own Gaelic grounds and that prioritise games of their own affiliated male players.

### 3.2. Media coverage

The issues surrounding lack of media exposure and respect and recognition of female athletes was an important concern of surveyed athletes. Respondents raised repeated concerns that they were not seen on TV, heard on the radio and generally not represented in the media to the same extent as their male counterparts. When asked whether media coverage was adequate currently, 75% either disagreed or strongly disagreed. However, there was recognition by some respondents that media coverage of women's sport was improving, as the following quotation indicates:

“While there has been an increase of media coverage of women's sports and some apparently large sponsorships etc in the last couple of years women's sports generally still appears to be of much less value than men's in the general culture, position in news headlines, opportunities for training times etc etc.” Basketball (non-elite) Dublin

Respondents reported that coverage of their own games and of female athletes they like to follow was inadequate, and considerably less than coverage of male athletes. They noted that finding reports, updates and basic coverage of women's games was a task. Players followed competitions by downloading certain apps or watching poor quality live streams from clubs' Facebook pages. They were not able to rely on mainstream media to provide coverage of games, either at all or of decent quality, especially games outside of senior finals or semi-finals. While some players acknowledged the importance and the success of broadcaster TG4's^3^ coverage of the LGFA championship and the AFL^4^ and that it had increased exposure of women in sport and had an overall positive impact, others highlighted their frustrations with the scarcity of media coverage of female sport, evident in the following statement;

“Irish women's soccer team were playing a game recently. Only to be watched on the RTÉ player. Meanwhile a men's soccer match was televised (not an Irish team). Our NATIONAL soccer team was not televised in this case, which we found ridiculous.”—Ladies Gaelic Football (non elite) Galway

Due to a lack of live sport during the pandemic, survey respondents felt that there was a greater appetite for sport of all types. There were limitations on sporting coverage of all sport throughout the pandemic as there was little to no live sporting events. Broadcasters like TG4 broadcast archival footage of past finals and other major games to fill the gap the absence of live sport left in their schedules (9). Despite the fact that women's competitions were more disrupted than men's, some respondents believed that respect, and interest in women's sport had increased in certain circumstances. The following comment highlights respondents' perception that media exposure of women's sport is important to players and critical to the growth of women's sports

“Having more games live streamed hugely improved access to wider audiences. Also, getting games on RTÉ really helped, as the analysis and quality of video was very good. Also, companies are now feeling embarrassed to be only giving money to men's sports, and more employees and customers are talking about it.”—Camogie (elite) Munster

While respondents agreed (47%) or strongly agreed (6%) that women's sport was in a more positive position prior to the pandemic, when asked about media coverage for women in sport at the time of the survey and prior to the pandemic, only 1% of respondents strongly agreed that it was adequate. Overall, the findings of the survey clearly suggest a concern among athletes that media coverage for women in sport needs to be significantly improved.

### 3.3. Funding

Funding was another issue raised by survey respondents. Concerns were expressed among respondents regarding a perceived lack of parity between male and female athletes and a lack of respect and recognition in clubs and counties for their female teams. Athletes noted that while this was evolving, continued pressure needed to be applied to clubs and county boards to ensure equal funding was provided for women's teams. Athletes noted, in relation to sponsorship of teams and players, that there was increasing external societal pressure on companies and organisations to support both male and female teams/athletes equally. However, overall 75% of respondents disagreed or strongly disagreed that women's sport in Ireland currently has sufficient sponsorship or support. Respondents also connected a lack of media coverage of women's sport with a lack of funding, as the response below indicates:

“Not enough funding, media coverage, but also girls can’t get off work for matches….. but the boys can; camogie matches, and training has to fit around hurling schedules; hard to get neutral pitches for games; good [coaches] want to train boys/men not girls/ladies. Hard to get sponsorship.”—Camogie (non-elite) Clare

Nonetheless, despite these challenges, some respondents felt positively about funding in the future for women's sport in Ireland and that change in terms of support and financing of teams was been driven, in part, by the pandemic and its role as an exposing force of the challenges and inequalities experienced by women participating in sport. These responses also reflected Irish government decisions in response to the pandemic to increase funding to sporting organisations and the debate this engendered regarding the need to put funding for women's sports on an equal footing with their male counterparts:

“The female GAA players receiving equal funding to men has been a major breakthrough and provides hope for other women's sports.”—Ladies Gaelic Football (elite) Donegal

This athlete is here referring to the announcement made by the Irish Government through Minister of State for Sport Jack Chambers in May 2021, that “parity of esteem and equality when it comes to funding” would be an Irish Government priority going forward (10).

### 3.4. Longer-term consequences of the pandemic

Respondents noted that the pandemic served to highlight that women's sport deserved to be given comparable recognition to that received by men's sports, and thereby acted as an exposing force of existing inequalities in this regard. Out of the 194 respondents, 68% answered “yes” when asked “As we emerge from the pandemic do you believe women's sport (its progression and support) will be in a better position?” For some, their reasoning was that women's sport was on the way to getting greater recognition and respect in society:

“Hopefully people will start to recognise the work is equally put in by men and women and women deserve equal rights and recognition”—Camogie (non-elite) Dublin

However, a major concern cited by survey respondents was the stagnation of progress for women in sport during the pandemic, particularly during the lockdown period and the subsequent lack of coverage given. Respondents expressed a fear that advancements made prior to the pandemic could be lost. Many noted the great achievements for women in sport prior to the pandemic, identifying the high attendances at All Ireland finals in both Ladies Gaelic football (56,114 in 2019) and Camogie (24,730 in 2019) in Croke Park and the 20 × 20^5^ campaign in particular. A major concern was that when sport returned women would be in a subordinate position once again and that major efforts will need to be made to ensure women and girls are encouraged to return to sport. However, some athletes had high hopes that the pandemic awakened people to the importance of sport and its centrality to communities and the importance of parity of funding for sports engaged in by both male and female athletes.

## 4. Conclusion

The COVID-19 pandemic in Ireland has been a challenging and revealing period with regard to sport, acting as an “exposing force” of the existing inequitable structures for both the support and coverage of women's sport. However, such challenging periods can also offer opportunities to learn, progress and respond more effectively to issues of concern, including with regard to the position of women's sport. The fact that 194 responses to the survey were received, which was live for less than a month, demonstrates that female athletes themselves are keen to share their experiences and opinions and to see progress in their sport. This suggests, perhaps, that the opportunity to express and share these viewpoints and concerns is not always available to them; or if it is, it is not adequately engaged with.

Creating parity of respect and recognition should, and will, continue to be a priority for women's sport. It is evident from the survey responses that female athletes are very aware of the coverage of women's sports, both in terms of its quality and quantity. They have communicated through their responses that greater media coverage of their sport elevates people's appreciation of women's sport and is an important aspect of gaining greater respect and recognition; this in turn will assist in addressing other concerns expressed by athletes, particularly in relation to inadequate sponsorship, funding, and access to facilities. A central issue that recurred in responses to the survey was a concern among female athletes that they were regarded effectively as second-class citizens when it came to access to facilities and resources. Following the pandemic a number of developments have occurred that may in time significantly benefit women's sport in Ireland. Firstly, the unequal funding of women's and men's sports was highlighted and the Irish government has increased significantly funding made available to women's sport as a result. In July 2021 Minister of State for Sport Jack Chambers announced €4 m in funding for the Sport Ireland Women in Sport programme (which funds women's sports in Ireland), a 33% increase on the 2019 allocation (11). This funding has increased still further in the 2023 budget which announced an overall record €52 million funding package dedicated to sport in Ireland, including funding to “support a number of initiatives aimed at improving the profile and visibility of female athletes across all sports, with specific support for women in football and women in rugby programmes” (12). Furthermore, to address the inequality between male and female athletes engaged with Gaelic games, significant moves have been made to amalgamate the LGFA and CA with the GAA and thereby ensure entitlement to equality of status for all athletes, including access to facilities, regardless of gender. In December 2020, the Gaelic Players Association (GPA) and Women's Gaelic Players Association (WGPA) agreed to merge the organisations into one body (13). This was followed in March 2022 by the decision by delegates at the Ladies Gaelic Football Association (LGFA) Congress to vote in favour of integrating with the GAA and Camogie Association (14). A process is now ongoing to facilitate this merger, with former President of Ireland, Mary McAleese as independent chairperson of the integration process between the three groups (15). While increased funding and the merger of these representative associations may have happened in time, these developments are unlikely to have happened as quickly as they did were it not for the “exposing force” of the COVID-19 pandemic of the unequal treatment of women's sport in Ireland.

## Data Availability

The raw data supporting the conclusions of this article will be made available by the authors, without undue reservation.

## Footnotes

^1^Among the safeguards were that respondents were not asked to identify their specific club, just the county or province they played in; questions were related to the respondents' experience as opposed to their identity; they were not asked to fill in any contact details; and they were informed that the survey was anonymous on the landing page of the survey.

^2^As part of comprehensive ‘Return to Play’ protocols issued by governing bodies of sport, all clubs were expected to appoint COVID-19 officers tasked with facilitating safe participation in sport and reducing the risk of spread of the coronavirus. The COVID-19 officer was responsible for ensuring safe environments for training and play through regular risk assessment and communication as well as tracking attendance at training and club events in the event of a need to trace contacts of people who acquire the illness. The LGFA, CA and GAA partnered together to establish these protocols – further information on these protocols is available here: https://ladiesgaelic.ie/wp-content/uploads/2021/04/Updated-Safe-Return-to-Gaelic-Games-Club-March-2021.pdf

^3^TG4 is an Irish-language terrestrial broadcaster. In 2021, TG4 broadcast 63 live games in the 2021 TG4 All-Ireland Championships of Ladies Gaelic Football [https://www.tg4.ie/en/information/press/press-releases/2021-2/lgfa-and-tg4-confirm-live-coverage/].

^4^AFL—Australian Football League.

^5^20×20 was an Irish government-led campaign aimed at creating a measurable cultural shift in the presentation and perception of Women’s sport in Ireland. It was a broad-based approach with three key metrics in mind to boost media coverage, attendance at key events and participation, each by 20 per cent between October 2018 and October 2020.

## References

[1] Mullally U. Covid-19 has exposed the serious injustices of Irish society. *Irish Times*, August 10 (2020). Available at: https://www.irishtimes.com/opinion/una-mullally-covid-19-has-exposed-the-serious-injustices-of-irish-society-1.4325936 (Accessed January 10, 2022)

[2] McElwee M Tomas F Garry T Brown O. Revealed: the true scale of how women's sport was left behind in lockdown. *The Telegraph*, March 23 (2021). Available at: https://www.telegraph.co.uk/sport/2021/03/23/revealed-true-scale-womens-sport-left-behind-lockdown/ (Accessed December 10, 2022).

[3] Pavlidis A Rowe D. The sporting bubble as gilded cage: gendered professional sport in pandemic times and beyond. M/C J. (2021) 24:1. 10.5204/mcj.2736

[4] Bowes A Lomax L Piasecki J. The impact of the COVID-19 lockdown on elite sportswomen. Manag Sport Leis. (2022) 27(6):513–29. 10.1080/23750472.2020.1825988

[5] Parry KD Clarkson BG Bowes A Grubb L Rowe D. Media framing of women’s football during the COVID-19 pandemic. Commun Sport. (2023) 11(3):592–615. 10.1177/21674795211041024 37520793 PMC9014347

[6] Osborne CA Skillen F. Women in sports history: the more things change, the more they stay the same? Sport Hist. (2020) 40(4):411–33. 10.1080/17460263.2020.1835707

[7] Rouse P. Off The Ball|History of Sport|‘Denounced, Belittled, Trivialised’—Women’s Presence in Sport. YouTube (2020). Available at: https://www.youtube.com/watch?v=aBxA3y_wyrk&ab_channel=OffTheBall (Accessed July 17, 2021).

[8] Madden P. COVID-19 officer roles & responsibilities. *Club Force* (2020). Available at: https://clubforce.com/latest-news/covid-19-officer-roles-responsibilities/ (Accessed April 17, 2023)

[9] Moran S. Golden memories to the fore as TG4 sift the GAA archives Epochal hurling titles for Galway and Offaly and Kerry’s four-in-a-row rare highlights in a dark era. *Irish Times*, June 22 (2020). Available at: https://www.irishtimes.com/sport/gaelic-games/golden-memories-to-the-fore-as-tg4-sift-the-gaa-archives-1.4284860 (Accessed May 15, 2023)

[10] RTÉ. Female GAA players set for equality in funding. May 10 (2021). Available at: Available at: https://www.rte.ie/sport/gaa/2021/0510/1217994-female-gaa-players-set-for-equality-in-funding/ (Accessed February 15, 2023).

[11] Cormican E. 33% increase in funding for women in sport to be announced today. *Irish Examiner*, July, 1 (2021). Available at: https://www.irishexaminer.com/sport/othersport/arid-40326635.html (Accessed March 10, 2023)

[12] Irish Government News Service. Major €52 million funding boost for Sports to provide for ongoing recovery of sector and support continued participation in sport and physical activity (2022). Available at: https://merrionstreet.ie/major_52_million_funding_boost_for_sports_to_provide_for_ongoing_recovery_of_sector_and_support_continued_participation_in_sport_and_physical_activity.html (Accessed March 11, 2023)

[13] Fogarty J. GPA and WGPA vote to merge into one association. *Irish Examiner*, 14 December (2020). Available at: https://www.irishexaminer.com/sport/gaa/arid-40190814.html (Accessed February 5, 2023)

[14] RTÉ. LGFA votes for GAA integration at congress. 4 March (2022). Available at: Available at: https://www.rte.ie/sport/womens-football/2022/0304/1284521-lgfa-votes-for-gaa-integration-at-congress/ (Accessed 10 March 2023).

[15] McGuire K. Former president of Ireland Mary McAleese to chair integration process between GAA, Camogie Association and LGFA. *scoreline.ie* (2022). Available at: https://scoreline.ie/former-president-of-ireland-mary-mcaleese-to-chair-integration-process-between-gaa-camogie-association-and-lgfa/ (Accessed March 10, 2023)

[16] Crosson S Free M. ‘This too shall pass’: gaelic games, Irish Media, and the COVID-19 lockdown in Ireland. In: Krieger J Henning A Dimeo P Pieper LP, editors. Time out: National perspectives on sport and the COVID-19 lockdown. Champaign, IL: Common Ground (2021). p. 289–306.

[17] Crosson S Free M. ‘Celebrate this victory in a sensible manner’: irish Media and the restart of gaelic games in Ireland. In: Krieger J Henning A Dimeo P Pieper LP, editors. “Restart” sport after the COVID-19 lockdown. Champaign, IL: Common Ground (2022). p. 33–49.

